# Main results of the Ouabain and Adducin for Specific Intervention on Sodium in Hypertension Trial (OASIS-HT): a randomized placebo-controlled phase-2 dose-finding study of rostafuroxin

**DOI:** 10.1186/1745-6215-12-13

**Published:** 2011-01-14

**Authors:** Jan A Staessen, Lutgarde Thijs, Katarzyna Stolarz-Skrzypek, Antonella Bacchieri, John Barton, Ezio degli Espositi, Peter W de Leeuw, Mirosław Dłużniewski, Nicola Glorioso, Andrzej Januszewicz, Paolo Manunta, Viktor Milyagin, Yuri Nikitin, Miroslav Souček, Chiara Lanzani, Lorena Citterio, Mario Timio, Andrzej Tykarski, Patrizia Ferrari, Giovanni Valentini, Kalina Kawecka-Jaszcz, Giuseppe Bianchi

**Affiliations:** 1 The Studies Coordinating Centre, Division of Hypertension and Cardiovascular Rehabilitation, Department of Cardiovascular Research, University of Leuven, Leuven, Belgium; 2 The Department of Epidemiology, Maastricht University, Maastricht, The Netherlands; 3 The First Department of Cardiology and Hypertension, Jagiellonian University Medical College, Kraków, Poland; 4 Sigma Tau Industrie Farmaceutiche Riunite, Pomezia, Italy; 5 Cardiac Research Department, Portiuncula Hospital, Galway, Ireland; 6 Unità di Valutazione dell' Efficacia Clinica, Ospedale San Maria delle Croci, Ravenna, Italy; 7 Department of Internal Medicine, Maastricht University Medical Centre, Maastricht, The Netherlands; 8 Department of Cardiology, Postgraduate Medical School, Warsaw, Poland; 9 Hypertension Centre, University of Sassari, Sassari, Italy; 10 Department of Internal Medicine and Hypertension, Medical Academy Warsaw, Warsaw, Poland; 11 Divisione di Nefrologia Dialisi e Ipertensione, Ospedale San Raffaele, Dipartimento di Scienze e Techologie Biomediche, Universitá Vita-Salute, Milan, Italy; 12 Department of Internal Medicine, Smolensk State Medical Academy, Smolensk, Russian Federation; 13 Institute of Internal Medicine, Siberian Branch of the Russian Academy of Medical Sciences, Novosibirsk, the Russian Federation; 14 Department of Internal Medicine 2, Saint Anna Hospital, University of Brno, Brno, The Czech Republic; 15 Department of Internal Medicine, Nephrology, and Dialysis, Hospital San Giovanni Battista, Perugia, Italy; 16 Department of Hypertension, School of Medicine, Poznan, Poland; 17 Prassis Research Institute, Settimo Milanese, Milan, Italy

## Abstract

**Background**

The Ouabain and Adducin for Specific Intervention on Sodium in Hypertension (OASIS-HT) Trial was a phase-2 dose-finding study of rostafuroxin, a digitoxygenin derivative, which selectively antagonizes the effects of endogenous ouabain (EO) on Na^+^,K^+^-ATPase and mutated adducin. Rostafuroxin lowered blood pressure (BP) in some animal models and in humans.

**Methods**

OASIS-HT consisted of 5 concurrently running double-blind cross-over studies. After 4 weeks without treatment, 435 patients with uncomplicated systolic hypertension (140-169 mm Hg) were randomized to rostafuroxin (0.05, 0.15, 0.5, 1.5 or 5.0 mg/d) or matching placebo, each treatment period lasting 5 weeks. The primary endpoint was the reduction in systolic office BP. Among the secondary endpoints were diastolic office BP, 24-h ambulatory BP, plasma EO concentration and renin activity, 24-h urinary sodium and aldosterone excretion, and safety. ANOVA considered treatment sequence (fixed effect), subjects nested within sequence (random), period (fixed), and treatment (fixed).

**Results**

Among 410 analyzable patients (40.5% women; mean age, 48.4 years), the differences in the primary endpoint (rostafuroxin minus placebo) ranged from -0.18 mm Hg (*P *= 0.90) on 0.15 mg/d rostafuroxin to 2.72 mm Hg (*P *= 0.04) on 0.05 mg/d. In the 5 dosage arms combined, the treatment effects averaged 1.30 mm Hg (*P *= 0.03) for systolic office BP; 0.70 mm Hg (*P *= 0.08) for diastolic office BP; 0.36 mm Hg (*P *= 0.49) for 24-h systolic BP; and 0.05 mm Hg (*P *= 0.88) for 24-h diastolic BP. In the 2 treatment groups combined, systolic (-1.36 mm Hg) and diastolic (-0.97 mm Hg) office BPs decreased from week 5 to 10 (*P *for period effect ≤0.028), but carry-over effects were not significant (*P *≥ 0.11). All other endpoints were not different on rostafuroxin and placebo. Minor side-effects occurred with similarly low frequency on rostafuroxin and placebo.

**Conclusions**

In 5 concurrently running double-blind cross-over studies rostafuroxin did not reduce BP at any dose.

**Trial Registration**

ClinicalTrials (NCT): NCT00415038

## Background

**Figure 1:**
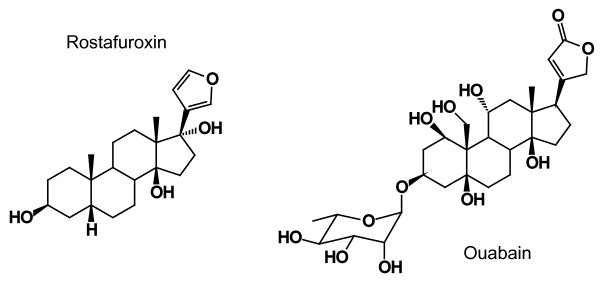
**Chemical structure of rostafuroxin and ouabain**.

Rostafuroxin (17β-[3-furyl]-5β-androstan-3β,14β,17α-triol; PST2238) is a digitoxygenin derivative (Figure 1), which selectively displaces ouabain from the Na^+^,K^+^-ATPase receptor [1,2]. Rostafuroxin has been developed in an attempt to unravel the contribution of mutated adducin and endogenous ouabain in the pathogenesis of hypertension [3]. The compound lowered blood pressure in Milan hypertensive rats and humans [4]. The Ouabain and Adducin for Specific Intervention on Sodium in Hypertension (OASIS-HT) Trial is a phase-2 dose finding study, which has been conducted at multiple centers in Europe [3]. The primary objective of this double-blind trial was to identify the minimal daily dose at which rostafuroxin in patients with uncomplicated hypertension would reduce blood pressure significantly more than placebo. The present paper summarizes the effects of rostafuroxin on blood pressure and sodium homeostasis and reports on drug safety.

## Methods

### Overview of the design of the trial

**Figure 2:**
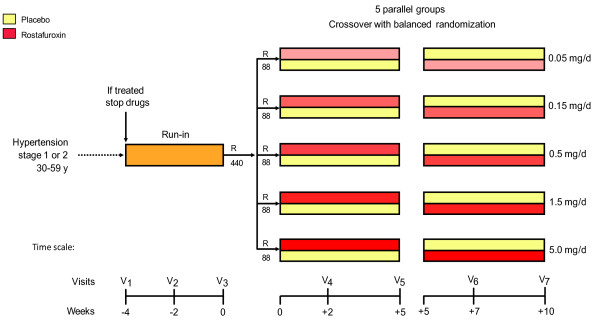
**Diagrammatic representation of the protocol. Numbers indicate patients projected to be enrolled**.

The protocol of the OASIS-HT trial (registration number http://clinicaltrial.gov, NCT00415038) has been published in detail elsewhere [3]. OASIS-HT was conducted according to rules of good clinical practice [5] at 39 European centers, which all received approval from the competent Ethics Committees and the National Regulatory Authorities. OASIS-HT was an early phase-2 dose-finding study (Figure 2). After a run-in period of 4 weeks, eligible patients were randomized to one of 5 oral doses of rostafuroxin (0.05, 0.15, 0.5, 1.5 or 5.0 mg/d). Each dose was compared to placebo in a double-blind cross-over experiment with balanced randomization. Treatment was initiated with the active drug and continued with placebo or vice versa. Each double-blind period lasted 5 weeks with an intermediate visit at 2 weeks and a final visit 3 weeks later. OASIS-HT was therefore a combination of 5 concurrent cross-over studies, one for each dose of rostafuroxin to be studied (Figure 2). The total duration of the study, including the 4-week run-in period, was 14 weeks. The wide range of doses used in OASIS-HT was based on preclinical studies that showed rostafuroxin inhibited ouabain-mediated actions at doses or concentrations that were approximately 10-fold lower than those required for antagonizing the effects of mutated adducin [2,6].

### Inclusion and exclusion criteria

Women and men, aged 30-59 years, with stage I or II hypertension according to the 2003 European guidelines [7] without any associated complications were eligible. At the screening visit, they had to be untreated or on treatment with only one drug or a single fixed combination tablet containing no more than 2 antihypertensive agents. At the screening visit, systolic blood pressure had to range from 140 to 169 mm Hg, irrespective of treatment status. At screening, patients gave written informed consent and those on treatment had their antihypertensive drugs discontinued. Two weeks later, while the patients were untreated, their systolic blood pressure had to remain above 140 mm Hg. At the end of the run-in period, 4 weeks after the screening visit, the untreated systolic blood pressure had to range from 140 to 169 mm Hg. In addition to hypertension, patients had to have less than 3 other risk factors, as defined by the European guidelines [7]. Women with childbearing potential had to apply an adequate contraceptive technique.

The exclusion criteria included a 24-h urinary Na^+ ^excretion exceeding 200 mmol at the end of the run-in period, malignant or secondary hypertension, disturbances of cardiac rhythm or conductance, electrocardiographic evidence of left ventricular hypertrophy, a history of myocardial infarction within 6 months of randomization, heart disease requiring treatment, a serum creatinine concentration higher than 1.3 mg/dL, microalbuminuria, gastrointestinal diseases which might influence the absorption or hepatic clearance of rostafuroxin, overt or medically-treated diabetes, a body mass index exceeding 30 kg/m^2^, mental disorders, and substance abuse.

### Randomization

Randomization followed a balanced incomplete block design. Each center was randomly allocated to one or more incomplete blocks. Within blocks, patients were randomly assigned to 4 or 6 of the 10 possible sequences of rostafuroxin (5 doses) and placebo (either preceding or following the active drug). A computerized procedure generating random numbers was used for randomization. Centers opted whether they would use blocks of study medication for 4 or 6 patients. At the end of the run-in period, the study manager at the coordinating office (Leuven, Belgium) checked all entry and exclusion criteria and after stratification for center randomized eligible patients to one of 5 doses of rostafuroxin (Figure 2). The first patient was randomized on April 6, 2005, and the last on March 20, 2007.

### Treatment

During the run-in period, the investigators informed patients on the life style changes to be implemented, including cessation of smoking, moderation of excessive alcohol intake, regular physical activity, weight reduction in overweight patients, and a reduction of dietary sodium intake to 120-160 mmol/d. A 24-h urinary sodium excretion at the end of the run-in period exceeding 200 mmol was an exclusion criterion. After randomization, in a cross-over design, patients took one of the 5 doses or rostafuroxin or matching placebo for 5 weeks and during the next 5 weeks switched to the alternative study medication. Short visits were scheduled 2 weeks after the initiation of each course of double-blind medication and visits with an extensive evaluation took place 3 weeks later at the end of each double-blind treatment period.

Uncontrolled hypertension was a blood pressure of at least 180 mm Hg systolic or 110 mm Hg diastolic on office measurement, confirmed within one week after the initial measurement. Patients with uncontrolled hypertension during the first treatment period were immediately withdrawn. Orthostatic hypotension was a drop in systolic blood pressure by at least 40 mm Hg on standing up from a sitting position. Patients experiencing orthostatic hypertension during the first treatment period were crossed over to the second treatment period. Patients with uncontrolled hypertension or orthostatic hypotension during the second treatment period proceeded immediately to the end-of-study evaluation.

Patients took their study medication around 8 AM before breakfast. Clinic visits were scheduled within 8 hours of drug intake. Depending on local conditions, each center standardized the time interval between the intake of the study medication and the measurement of the clinic blood pressure. On the days before the end-of-period visits 5 and 7 (Figure 2), the patients fasted overnight. On these days, they took their study medication at the examination center after blood sampling and shortly before the start of the ambulatory blood pressure recording and the 24-h urine collection. Patients returned unused capsules at each visit.

### Blood pressure measurement

At the screening visit, the investigator determined the appropriate cuff size for each patient. If arm circumference was less than 32 cm, a standard cuff with a bladder of 12 × 22 cm was applied. For arms with larger circumference, cuffs had a bladder size of 15 × 31 cm. If at the screening visit, the differences in systolic and diastolic blood pressures between both arms were less than 10 mm Hg, all blood pressure readings throughout the trial were obtained at the non-dominant arm. Otherwise, the arm giving the highest blood pressure readings was used.

At each visit, after the patients had rested for 5 minutes in the sitting position, the observer measured the office blood pressure 5 times consecutively at the brachial artery, using the validated oscillometric Omron *705IT *recorder (Omron Healthcare Europe BV, Nieuwegein, The Netherlands). The average of the last 3 readings was used to determine eligibility and for statistical analysis. Immediately after the 5 blood pressure measurements with the patient in the sitting position, the observer obtained 2 blood pressure readings while the patient was standing. These 2 standing measurements were averaged to determine the occurrence of orthostatic hypotension. Throughout the trial, each patient was followed up using the same Omron *705IT *device applied to the same arm by means of equally sized cuffs. To detect irregularities, the quality of the blood pressure readings was monitored during the study with respect to digit and number preference [8].

At the end of the run-in period as well as at the end of the 2 double-blind treatment periods, patients underwent 24-h ambulatory blood pressure monitoring. Validated oscillometric SpaceLabs 90207 recorders (SpaceLabs Inc, Redmond, WA [9]) were programmed to obtain blood pressure readings at 15-minute intervals from 8 AM to 10 PM and at 30-minute intervals otherwise. Daytime and night-time were defined on the basis of the short fixed clock-time method [10] with intervals ranging from 10 AM to 8 PM and from midnight to 6 AM, respectively. Intra-individual means of the ambulatory measurements were weighted by the time interval between successive readings [11].

#### Other measurements

Venous blood was collected at baseline and at the end of each treatment period. Immediately after blood sampling, the patient provided a fresh urine specimen, took the study medication and started a 24-h urine collection. Daytime and night-time urines were collected in separate containers. In addition to the routine hematological and biochemical measurements for safety, measurements included plasma renin activity, the plasma concentration of endogenous ouabain, the 24-h urinary excretion of sodium, potassium, aldosterone and creatinine, and semi-quantitative dipstick tests on a fresh urine sample. Plasma renin activity, plasma ouabain and urinary aldosterone were measured centrally at the Divisione di Nefrologia Dialisi e Ipertensione, Ospedale San Raffaele (Milan, Italy). At baseline and at the end of each treatment period, patients also underwent a standard 12-lead ECG recording.

#### Statistical analyses

The primary efficacy measure was the sitting systolic blood pressure measured at the office. With significance set at 5% and power at 90%, approximately 440 patients, 88 in each crossover arm, had to be randomized to detect within each arm of the trial a blood pressure difference of 8 mm Hg between rostafuroxin and placebo, assuming a standard deviation of 15 mm Hg and a drop-out rate of 10%.

Statistical analyses were performed according to the intention-to-treat principle with SAS software, version 9.2 (SAS Institute Inc, Cary, NC). The analysis set included all patients who had at least one evaluation available for each of the 2 cross-over periods. In case of missing data, the last observation within each period was carried forward. Patients who withdrew from the study during the first treatment period because of uncontrolled hypertension contributed to the statistical analysis with the last blood pressure for the first period; for the second period they were assigned the average blood pressure on the treatment, which they should have taken during the second period.

Measurements with a skewed distribution were normalized by a logarithmic transformation. Sodium and potassium excretions were standardized to 24 hours. The effects of rostafuroxin on blood pressure and other measurements were evaluated using analysis of variance with the treatment sequence (fixed effect), subjects nested within sequence (random effect), period (fixed effect) and treatment (fixed effect) entered as covariables. Measurements obtained at the end of each treatment period were used as dependent variables. Analyses were performed in each dosage arm separately and in all dosage arms combined. The hypothesis of no sequence effect in the ANOVA model was tested to check for carry-over effects. In sensitivity analyses, we analyzed the blood pressure changes from baseline to week 5, using Student's t-test for paired and unpaired observations. We also adjusted the ANOVA for 24-h urinary sodium excretion and did a separate analysis in patients whose 24-h urinary sodium excretion was less than 200 mmol at both weeks 5 and 10.

## Results

### Subject characteristics

**Figure 3:**
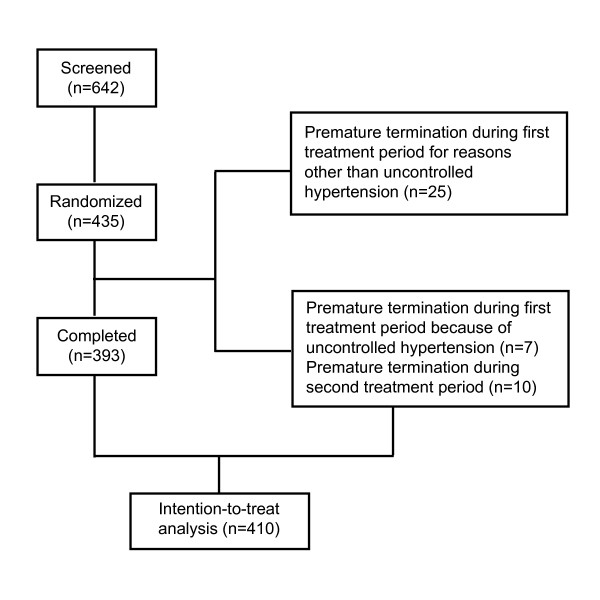
**Trial profile**.

Of the 642 screened patients, 435 (67.8%) met the entry criteria and were randomized. Twenty-five patients were not included in the analysis because they prematurely terminated the trial in the first treatment period (Figure 3). The reasons for premature termination were withdrawal of consent (n = 16), adverse events other than uncontrolled hypertension (n = 4), intake of concomitant medication prohibited by the protocol (n = 3), loss to follow-up (n = 1), and undocumented reasons (n = 1).

**Figure 4:**
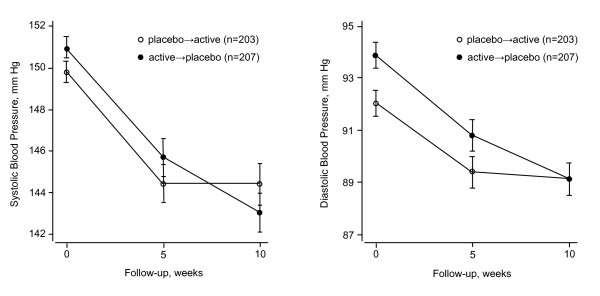
**Sitting systolic and diastolic blood pressures at randomization and at the end of each treatment period**. Means and standard errors are presented for the 5 dosage groups combined. Open circles indicate the patients receiving placebo during the first treatment period and rostafuroxin during the second treatment period. Closed circles indicate the patients receiving rostafuroxin during the first treatment period and placebo during the second treatment period.

The baseline characteristics of the 166 women and 244 men included in the present analysis were similar among all dosage and treatment arms (Table 1) with the exception of sitting diastolic blood pressure, which was 1.9 mm Hg lower (92.0 *vs *93.9 mm Hg; *P *= 0.011; Figure 4) in the placebo-to-active arm (n = 203) as compared to the active-to-placebo arm (n = 207). Mean age (± SD) was 48.4 ± 7.9 years. Conventional blood pressure averaged 150.4 ± 7.6 mm Hg systolic and 93.0 ± 7.3 mm Hg diastolic. For the 24-h blood pressure, these values were 137.3 ± 10.9 mm Hg and 85.8 ± 8.1 mm Hg, respectively. Seventy-two (17.6%) patients were current smokers and 203 (49.5%) reported alcohol intake.

**Table 1:** Baseline characteristics by dosage and treatment arm

Characteristic	0.05 mg/d		0.15 mg/d		0.5 mg/d		1.5 mg/d		5.0 mg/d	
	
	P/R (N = 43)	R/P (N = 44)	P/R (N = 44)	R/P (N = 44)	P/R (N = 41)	R/P (N = 44)	P/R (N = 44)	R/P (N = 44)	P/R (N = 44)	R/P (N = 43)
Mean (± SD) of baseline characteristic										
Age, years	48.1 ± 7.8	50.4 ± 7.4	46.8 ± 8.6	48.2 ± 8.0	48.8 ± 7.2	49.7 ± 7.5	49.0 ± 6.7	48.5 ± 8.5	49.7 ± 7.9	44.9 ± 8.4
BMI, kg/m^2^	27.1 ± 2.7	27.6 ± 2.3	26.4 ± 2.7	26.9 ± 2.2	26.3 ± 2.7	27.0 ± 2.5	25.9 ± 3.0	26.9 ± 2.4	25.8 ± 2.5	26.7 ± 2.7
Pulse rate, beats/min	77.0 ± 10.8	77.6 ± 10.3	75.8 ± 12.8	74.4 ± 9.8	75.2 ± 11.0	76.8 ± 12.3	74.2 ± 11.8	80.5 ± 11.5	75.8 ± 13.0	77.3 ± 10.0
Systolic BP, mm Hg										
Office	148.7 ± 7.3	150.9 ± 7.4	148.0 ± 5.8	150.5 ± 8.4	150.4 ± 7.6	152.5 ± 8.7	150.8 ± 7.6	151.1 ± 7.8	150.6 ± 6.7	149.4 ± 7.2
24-h ambulatory*	136.8 ± 9.5	137.3 ± 11.3	135.3 ± 10.4	134.9 ± 10.9	140.9 ± 11.0	137.4 ± 9.6	138.1 ± 11.2	137.8 ± 11.8	138.6 ± 11.4	136.2 ± 11.7
Daytime*	144.4 ± 9.9	143.7 ± 12.5	141.8 ± 10.4	142.3 ± 12.3	147.1 ± 12.7	142.9 ± 10.1	145.0 ± 13.6	144.5 ± 12.5	145.2 ± 12.6	142.2 ± 12.7
Night-time*	122.2 ± 11.9	123.5 ± 13.8	122.5 ± 14.8	119.9 ± 11.3	126.4 ± 12.2	125.6 ± 12.6	125.7 ± 11.7	123.5 ± 14.7	125.5 ± 13.0	122.8 ± 15.4
Diastolic BP, mm Hg										
Office	92.1 ± 7.6	93.0 ± 7.0	91.9 ± 7.2	92.4 ± 7.2	92.3 ± 6.9	95.9 ± 7.2	92.0 ± 7.9	94.2 ± 8.2	92.8 ± 6.4	93.9 ± 6.5
24-h ambulatory*	85.5 ± 7.9	84.1 ± 7.2	84.5 ± 7.6	84.2 ± 8.4	88.1 ± 9.4	87.6 ± 8.8	86.5 ± 7.3	85.6 ± 8.4	86.9 ± 7.0	85.0 ± 9.0
Daytime*	91.6 ± 8.2	89.7 ± 8.8	90.2 ± 7.5	90.2 ± 9.5	93.2 ± 10.9	92.3 ± 9.1	91.5 ± 8.8	91.0 ± 8.8	92.5 ± 8.3	89.6 ± 10.0
Night-time*	73.4 ± 9.7	72.7 ± 8.4	74.3 ± 10.8	72.3 ± 7.8	77.1 ± 9.5	78.4 ± 10.8	77.1 ± 8.6	74.7 ± 11.0	76.6 ± 7.7	74.2 ± 10.5

Number (%) with baseline characteristic										
Women	20 (46.5)	18 (40.9)	21 (47.7)	15 (34.1)	12 (29.3)	14 (31.8)	21 (47.7)	16 (36.4)	22 (50.0)	18 (41.9)
Caucasian	43 (100)	44 (100)	44 (100)	44 (100)	41 (100)	42 (95.5)	43 (97.7)	43 (97.7)	44 (100)	43 (100)
Previous AH treatment	14 (32.6)	14 (31.8)	22 (50.0)	20 (45.5)	18 (43.9)	24 (54.5)	23 (52.3)	22 (50.0)	18 (40.9)	18 (41.9)
Current smokers	5 (11.6)	7 (15.9)	6 (13.6)	9 (20.5)	6 (14.6)	8 (18.2)	5 (11.4)	7 (15.9)	14 (31.8)	12 (27.9)
Current alcohol intake	21 (48.8)	23 (52.3)	19 (43.2)	23 (52.3)	22 (53.7)	21 (47.7)	18 (40.9)	23 (52.3)	20 (45.5)	27 (62.8)

Values are means ± SD or number (percentage) of patients. N indicates the number of patients. Abbreviations: P/R, 5 weeks placebo followed by 5 weeks rostafuroxin; A/R, 5 weeks rostafuroxin followed by 5 weeks placebo; BP, blood pressure; AH, antihypertensive before the run-in period.

*Ambulatory blood pressure recordings were available in 383 patients.

### Blood pressure

The conventional systolic and diastolic blood pressures decreased (*P *≤ 0.0001) on average by 5.3 ± 12.0 mm Hg and 2.9 ± 8.1 mm Hg from baseline to week 5 (Figure 4). For the 24-h ambulatory systolic and diastolic blood pressures, these reductions averaged 1.5 ± 10.5 mm Hg (*P *= 0.0056) and 1.5 ± 6.6 mm Hg (*P *≤ 0.0001), respectively. However, in each of the 5 dosage arms, blood pressures were similar on rostafuroxin as compared to placebo (Tables 2 and 3). The between-treatment differences in the primary endpoint (active minus placebo treatment) ranged from -0.18 mm Hg (95% confidence interval [CI], -3.21 to 2.85; *P *= 0.90) on 0.15 mg/d rostafuroxin to 2.72 mm Hg (CI, 0.07 to 5.36; *P *= 0.04) on 0.05 mg/d (Table 2). In the 5 dosage arms combined, the between-treatment differences averaged 1.30 mm Hg (CI, 0.10 to 2.50; *P *= 0.03) for the systolic office blood pressure; 0.70 mm Hg (CI, -0.09 to 1.50; *P *= 0.08) for the diastolic office blood pressure; 0.36 mm Hg (CI, -0.67 to 1.38; *P *= 0.49) for the systolic 24-h blood pressure; and 0.05 mm Hg (CI, -0.61 to 0.72; *P *= 0.88) for the diastolic 24-h blood pressure. Similar results were obtained when the day- and night-time blood pressures were analyzed separately. No significant (*P *≥ 0.11) carry-over effect was detected in any of the dosage arms for any of the blood pressure endpoints. In the 2 treatment groups combined, the conventional systolic (-1.36 ± 12.4 mm Hg) and diastolic (-0.97 ± 8.3 mm Hg) blood pressures decreased slightly from week 5 to week 10, leading to a significant period effect (*P *= 0.028 and *P *= 0.018 respectively).

**Table 2:** Office blood pressure on treatment with placebo or different daily doses of rostafuroxin

		Mean level ± SD at end of treatment period		Mean difference (95% CI)	*P*-values		
		
	N	Placebo	Rostafuroxin	Rostafuroxin minus placebo	Treatment effect	Carryover effect	Period effect
Systolic pressure, mm Hg							
0.05 mg	83	142.0 ± 11.1	144.7 ± 12.2	2.72 (0.07 to 5.36)	0.044	0.352	0.229
0.15 mg	82	143.6 ± 13.5	143.3 ± 13.9	-0.18(-3.21 to 2.85)	0.905	0.897	0.364
0.5 mg	77	145.3 ± 13.8	145.9 ± 13.3	0.49 (-2.38 to 3.36)	0.732	0.326	0.770
1.5 mg	86	145.8 ± 13.7	146.8 ± 15.1	1.11 (-1.32 to 3.54)	0.366	0.828	0.723
5.0 mg	82	142.1 ± 12.1	144.5 ± 14.3	2.29 (-0.35 to 4.93)	0.089	0.667	0.038
All doses	410	143.7 ± 12.9	145.1 ± 13.8	1.30 (0.10 to 2.50)	0.034	0.963	0.028

Diastolic pressure, mm Hg							
0.05 mg	83	88.1 ± 8.3	89.9 ± 9.2	1.76 (0.01 to 3.51)	0.049	0.846	0.639
0.15 mg	82	88.7 ± 9.1	88.5 ± 9.6	-0.25 (-2.03 to 1.54)	0.786	0.596	0.296
0.5 mg	77	90.6 ± 8.8	90.6 ± 8.2	0.02 (-1.92 to 1.95)	0.986	0.112	0.905
1.5 mg	86	90.5 ± 9.5	91.5 ± 8.9	0.98 (-0.65 to 2.61)	0.235	0.580	0.027
5.0 mg	82	88.4 ± 8.1	89.4 ± 8.5	0.97 (-1.01 to 2.95)	0.333	0.431	0.163
All doses	410	89.3 ± 8.8	90.0 ± 8.9	0.70 (-0.09 to 1.50)	0.084	0.364	0.018

Mean differences were estimated using analysis of variance with sequence, subjects nested within sequence, and period and treatment group entered as covariables. Abbreviations: N = number of patients; SD, standard deviation; CI, confidence interval.

**Table 3:** 24-h ambulatory blood pressure on treatment with placebo and different daily doses of rostafuroxin

		Mean level ± SD at end of treatment period		Mean difference (95% CI)	*P*-values		
		
	N	Placebo	Rostafuroxin	Rostafuroxin minus placebo	Treatment effect	Carryover effect	Period effect
Systolic pressure, mm Hg							
0.05 mg	79	133.1 ± 10.8	134.1 ± 11.2	0.99 (-1.42 to 3.40)	0.417	0.398	0.324
0.15 mg	76	134.8 ± 13.3	134.7 ± 12.0	-0.07 (-2.73 to 2.58)	0.957	0.348	0.166
0.5 mg	72	137.0 ± 10.7	137.0 ± 10.8	0.08 (-2.27 to 2.43)	0.945	0.790	0.889
1.5 mg	80	136.7 ± 13.1	137.8 ± 10.7	1.11 (-1.04 to 3.26)	0.306	0.753	0.741
5.0 mg	76	136.3 ± 11.8	135.9 ± 12.9	-0.36 (-2.37 to 1.64)	0.719	0.854	0.696
All doses	383	135.6 ± 12.0	135.9 ± 11.6	-0.36 (-0.67 to 1.38)	0.493	0.926	0.860

Diastolic pressure, mm Hg							
0.05 mg	79	82.5 ± 8.3	83.2 ± 8.3	0.75 (-0.92 to 2.41)	0.374	0.611	0.446
0.15 mg	76	83.4 ± 9.0	83.1 ± 9.3	-0.29(-2.03 to 1.45)	0.743	0.467	0.155
0.5 mg	72	86.2 ± 8.4	85.2 ± 9.0	-1.01(-2.59 to 0.58)	0.210	0.459	0.630
1.5 mg	80	84.9 ± 9.3	85.4 ± 7.7	0.56 (-0.66 to 1.77)	0.365	0.900	0.559
5.0 mg	76	84.7 ± 8.5	84.8 ± 8.6	0.18 (-1.11 to 1.46)	0.785	0.687	0.677
All doses	383	84.3 ± 8.8	84.3 ± 8.6	0.05 (-0.61 to 0.72)	0.882	0.457	0.971

Mean differences were estimated using analysis of variance with sequence, subjects nested within sequence, and period and treatment group entered as covariables. Abbreviations: CI, confidence interval; N = number of patients; SD, standard deviation.

### Excretion of electrolytes

At baseline urinary volume averaged 1502 ± 574 mL/24 h and the urinary sodium and potassium excretion 152.8 ± 55.7 mmol/24 h and 62.7 ± 35.2 mmol/24 h, respectively. The sodium and potassium excretion rates were greater (*P *≤ 0.001) in daytime (115.0 ± 50.1 μmol/min and 50.3 ± 31.5 μmol/min) than nighttime urine collections (90.7 ± 52.3 μmol/min and 30.8 ± 26.1 μmol/min). Both the daytime (+12.6 ± 88.0 μmol/min; *P *= 0.0051) and nighttime (+17.4 ± 73.1 μmol/min; *P *< 0.0001) sodium excretion rates, but not the potassium excretion rates, increased significantly from baseline to week 5, but thereafter remained unchanged (*P *≥ 0.11).

The 24-h, day- and nighttime excretion rates of sodium and potassium were similar on placebo and active treatment in all 5 dosage arms. The between-treatment differences in the 5 dosage arms combined averaged -5.1 μmol/min (CI, -10.5 to 0.4; *P *= 0.071) for the 24-h sodium excretion and -0.2 μmol/min (CI, -2.6 to 2.2; *P *= 0.84) for the 24-h potassium excretion (Table 4). Carry-over effects were not statistically significant (*P *≥ 0.55).

**Table 4:** Urinary excretion rate of electrolytes, plasma ouabain concentration and plasma renin activity on placebo and on rostafuroxin

		Mean level at the end of treatment period		Mean difference (95% CI)	*P*-values		
		
	N	Placebo	Rostafuroxin	Active minus placebo	Treatment effect	Carryover effect	Period effect
Sodium (μmol/min)							
Whole day	386	122.3 ± 53.1	117.3 ± 50.4	-5.1 (-10.5 to 0.4)	0.071	0.546	0.256
Daytime	386	128.2 ± 69.8	126.3 ± 86.7	-1.9 (-10.9 to 7.0)	0.672	0.802	0.880
Nighttime	386	115.6 ± 87.4	107.6 ± 67.8	-8.0 (-16.4 to 0.4)	0.061	0.710	0.112

Potassium (μmol/min)							
Whole day	386	43.7 ± 22.1	43.5 ± 20.2	-0.2 (-2.6 to 2.2)	0.844	0.942	0.685
Daytime	386	49.5 ± 29.1	50.7 ± 38.4	1.2 (-2.8 to 5.2)	0.545	0.641	0.320
Nighttime	386	33.4 ± 27.3	32.3 ± 21.8	-1.1 (-4.2 to 2.0)	0.479	0.692	0.775

Sodium/potassium ratio							
Whole day	386	2.84 (0.99 to 7.74)	2.72 (0.96 to 8.13)	0.96 (0.90 to 1.01)	0.122	0.434	0.154
Daytime	386	2.64 (0.81 to 8.29)	2.53 (0.77 to 8.66)	0.96 (0.90 to 1.02)	0.196	0.490	0.273
Nighttime	386	3.46 (1.03 to 10.6)	3.34 (1.06 to 11.6)	0.97 (0.90 to 1.04)	0.327	0.093	0.314

Plasma ouabain (pmol/L)	357	155.4 (56.8 to 453)	161.6 (57.7 to 425.0)	1.04 (0.98 to 1.10)	0.192	0.997	0.369

Plasma renin activity (ng/mL/h)	374	0.57 (0.11 to 2.62)	0.57 (0.12 to 2.95)	1.00 (0.94 to 1.07)	0.948	0.087	0.672

Aldosterone excretion (nmol/24 h)	334	25.7 (5.96 to 130)	25.5(4.99 to 111)	0.99(0.90 to 1.09)	0.862	0.366	0.377

Mean levels at the end of the treatment period are arithmetic means ± SD or geometric means (95% confidence interval). Mean differences were estimated using analysis of variance with sequence, subjects nested within sequence, and period and treatment group entered as covariables. Mean differences are expressed as the difference of rostafuroxin minus placebo or the ratio of rostafuroxin to placebo for non-transformed and logarithmically transformed variables, respectively. Abbreviations: CI, confidence interval; N = number of patients.

### Plasma ouabain, plasma renin activity and urinary aldosterone excretion

At baseline the geometric means of plasma ouabain, plasma renin activity, and the urinary aldosterone excretion were 164.3 pmol/L (CI, 57.1 to 472.5), 0.58 ng/mL/h (CI, 0.12 to 2.81), and 26.3 nmol/24 h (CI, 4.63 to 150.1), respectively. These hormones did not change during follow-up and were similar on placebo and rostafuroxin in each of the 5 dosage arms (Table 4). The between-group ratios (active divided by placebo) in the 5 dosage arms combined were 1.04 pmol/L (CI, 0.98 to 1.10; *N *= 357; *P *= 0.192), 1.00 pmol/L (CI, 0.94 to 1.07; *N *= 374; *P *= 0.95) and 0.99 (CI, 0.90 to 1.09; *N *= 334; *P *= 0.86) for plasma ouabain, plasma renin activity and the 24-h urinary aldosterone excretion, respectively (Table 4).

### Safety

Among the 435 randomized patients, a total of 96 adverse events were reported on active treatment and 56 on placebo treatment. These events occurred in 64 and 50 patients, respectively (*P *= 0.14). Table 5 lists the adverse events that occurred in at least 3 patients. The following adverse events led to premature discontinuation of the trial: uncontrolled hypertension (3 on placebo and 6 on rostafuroxin), fatal ruptured aortic aneurysm (1 patient on placebo), metastatic cancer (1 patient on placebo), loss of consciousness (1 patient on rostafuroxin), increases in γ-glutamyltransferase and alkaline phosphatase activities (1 patient on placebo), nausea and bloated feeling (1 patient on placebo), and cough and skin irritation (1 patient on rostafuroxin). The hematological measurements and those reflecting renal and liver function were similar at baseline and follow-up (*P *≥ 0.10) and none were different on placebo and rostafuroxin (*P *≥ 0.18) with one exception. Hemoglobin was slightly lower on rostafuroxin than placebo (8.99 *vs *9.03 mmol/L; *P *= 0.049).

**Table 5:** Number of patients with adverse events on placebo and different daily doses of rostafuroxin

	Placebo	Rostafuroxin					
	**All**	**All**	**0.05 mg**	**0.15 mg**	**0.5 mg**	**1.5 mg**	**5.0 mg**
	
Total number of patients	419*	419†	84	84	81	87	83
	
Number with complaints							
Osteomuscular‡	4	3	1	1	0	0	1
Gastro-intestinal#	5	5	0	1	1	1	2
Dizziness	2	5	0	1	0	4	0
Dry mouth	1	4	1	0	0	1	2
Headache	8	14	3	4	2	2	3
High blood pressure	4	6	1	3	2	0	0
Otitis	1	2	0	1	1	0	0
Palpitations	1	3	0	0	1	0	2
Cutaneous§	0	4	2	1	0	1	0
Upper respiratory tract infections	5	8	5	1	0	0	2
Urinary abnormalities¶	0	5	2	0	2	1	0
Urinary tract infections	2	5	1	2	0	2	0

Values are number of subjects.

*16 subjects did not receive placebo, because they withdrew from the trial in the first treatment period, while on rostafuroxin.

†16 subjects did not receive rostafuroxin, because they withdrew from the trial in the first treatment period, while on placebo.

‡Bursitis or pain in back, neck, knees, or shoulders

#Distension, abdominal pain, dyspepsia, gastritis, gastroenteritis or nausea.

§Skin irritation, rash or dermatitis.

¶Proteinuria, glucosuria, or hematuria on semiquantitative dipstick test.

At baseline, heart rate averaged 76.2 ± 11.5 beats/min on conventional measurement (sitting) and 74.5 ± 8.5 beats/min on 24-h ambulatory measurement. The baseline values of the ECG intervals were 157.0 ± 24.9 msec, 85.3 ± 11.6 msec and 381.2 ± 40.8 msec, and 401.7 ± 52.5 msec for PR, QRS, QT and QT_c_, respectively. None of these electrocardiographic measurements changed during follow-up (*P *≥ 0.77) and all were similar on placebo and rostafuroxin (*P *≥ 0.083).

### Sensitivity analyses

There was a significant period effect with a slight, but significant reduction in the conventional blood pressure from week 5 until week 10 (see above). We therefore did an additional analysis including only blood pressures at baseline and at 5 weeks, according to a parallel-group design based on the first randomization (Figure 2). The results for the office and 24-h ambulatory blood pressures appear in Tables 6 and 7. The results of this parallel-group analysis confirmed those of the double-blind cross-over analysis.

**Table 6:** Office blood pressure at the end of the baseline period and at 5 weeks after randomization

		Mean level ± SD at end of period		Mean differences (95% CI)		*P*-value
	**N**	**Baseline**	**5 weeks**	**5 weeks minus** **baseline**	**Double difference** **(rostafuroxin minus placebo)**	

Systolic pressure, mm Hg						
Placebo	203	149.8 ± 7.2	144.5 ± 12.5	-5.35 (-6.97 to -3.74)		
Rostafuroxin						
0.05 mg	42	150.5 ± 7.3	144.5 ± 12.6	-6.00 (-9.86 to -2.13)	-1.20 (-6.28 to 3.87)	0.64
0.15 mg	40	150.5 ± 8.4	143.9 ± 12.4	-6.60 (-9.91 to -3.29)	-3.08 (-8.03 to 1.88)	0.22
0.5 mg	40	153.0 ± 8.7	147.4 ± 11.5	-5.65 (-9.42 to -1.88)	0.96 (-4.74 to 6.66)	0.74
1.5 mg	43	151.0 ± 7.9	147.4 ± 15.2	-3.68 (-7.87 to 0.51)	1.59 (-3.77 to 6.95)	0.56
5.0 mg	42	149.6 ± 7.1	145.3 ± 14.0	-4.31 (-8.55 to -0.07)	2.45 (-3.05 to 7.95)	0.38
All doses	207	150.9 ± 7.9	145.7 ± 13.2	-5.22 (-6.92 to -3.53)	0.13 (-2.21 to 2.47)	0.91

Diastolic pressure, mm Hg						
Placebo	203	92.0 ± 7.2	89.4 ± 8.7	-2.63 (-3.77 to -1.50)		
Rostafuroxin						
0.05 mg	42	92.9 ± 7.2	89.9 ± 8.3	-3.01 (-4.90 to -1.11)	0.52 (-2.65 to 3.70)	0.75
0.15 mg	40	92.3 ± 7.5	88.5 ± 8.5	-3.82 (-6.12 to -1.51)	-1.94 (-5.52 to 1.65)	0.28
0.5 mg	40	95.9 ± 6.6	92.0 ± 7.8	-3.96 (-6.67 to -1.25)	-1.20 (-5.32 to 2.92)	0.56
1.5 mg	43	94.2 ± 8.3	92.9 ± 9.4	-1.33 (-3.77 to 1.12)	-0.21 (-3.26 to 2.84)	0.89
5.0 mg	42	94.0 ± 6.5	90.7 ± 9.2	-3.33 (-6.32 to -0.35)	0.68 (-3.15 to 4.51)	0.72
All doses	207	93.9 ± 7.3	90.8 ± 8.7	-3.06 (-4.15 to -1.98)	-0.43 (-2.00 to 1.13)	0.59

Mean differences were estimated with a paired and unpaired t-tests for within-group and between-group differences, respectively. The double difference is the net treatment effect defined as the difference placebo minus baseline subtracted from the difference rostafuroxin minus baseline (parallel-group analysis). Abbreviations: N = number of patients; SD, standard deviation; CI, confidence interval.

**Table 7:** 24-h ambulatory blood pressure at the end of the baseline period and at 5 weeks after randomization

		Mean level ± SD at end of period		Mean differences (95% CI)		*P*-value
	**N**	**Baseline**	**5 weeks**	**5 weeks minus** **baseline**	**Double difference** **(rostafuroxin minus placebo)**	

Systolic pressure, mm Hg						
Placebo	189	137.8 ± 10.7	135.6 ± 12.2	-2.28 (-3.78 to -0.77)		
Rostafuroxin						
0.05 mg	39	137.3 ± 11.3	132.5 ± 11.2	-4.74 (-8.00 to -1.48)	-1.16 (-5.38 to 3.06)	0.59
0.15 mg	36	134.9 ± 10.9	137.3 ± 11.2	+2.36 (-0.62 to 5.34)	3.16 (-1.32 to 7.63)	0.16
0.5 mg	38	137.4 ± 9.6	136.7 ± 9.9	-0.76 (-4.44 to 2.92)	2.63 (-2.44 to 7.70)	0.30
1.5 mg	40	137.8 ± 11.8	138.0 ± 10.3	+0.21 (-3.10 to 3.51)	2.12 (-2.74 to 6.98)	0.39
5.0 mg	36	136.2 ± 11.7	135.6 ± 11.9	-0.52 (-4.32 to 3.27)	1.33 (-4.00 to 6.66)	0.62
All doses	189	136.8 ± 11.0	136.0 ± 11.0	-0.74 (-2.24 to 0.77)	1.54 (-0.59 to 3.66)	0.16

Diastolic pressure, mm Hg						
Placebo	189	86.2 ± 7.8	84.0 ± 8.7	-2.28 (-3.28 to -1.28)		
Rostafuroxin						
0.05 mg	39	84.1 ± 7.2	82.4 ± 8.4	-1.65 (-3.25 to -0.06)	1.62 (-0.74 to 3.97)	0.18
0.15 mg	36	84.2 ± 8.4	84.6 ± 9.1	+0.41 (-1.46 to 2.28)	1.62 (-1.23 to 4.47)	0.26
0.5 mg	38	87.6 ± 8.8	85.7 ± 8.3	-1.89 (-4.15 to 0.37)	0.68 (-2.65 to 4.01)	0.68
1.5 mg	40	85.6 ± 8.4	85.4 ± 7.4	-0.28 (-1.96 to 1.40)	1.64 (-1.21 to 4.50)	0.25
5.0 mg	36	85.0 ± 9.0	85.4 ± 8.4	+0.36 (-2.34 to 3.07)	2.85 (-0.86 to 6.57)	0.13
All doses	189	85.3 ± 8.4	84.7 ± 8.3	-0.63 (-1.52 to 0.26)	1.65 (0.31 to 2.98)	0.02

Mean differences were estimated with paired and unpaired t-tests for within-group and between-group differences, respectively. The double difference is the net treatment effect defined as the difference placebo minus baseline subtracted from the difference rostafuroxin minus baseline (parallel-group analysis). Abbreviations: N = number of patients; SD, standard deviation; CI, confidence interval.

If the ANOVA model used for the cross-over analysis of blood pressure was adjusted for 24-h sodium excretion, the mean difference (rostafuroxin all doses combined minus placebo) was 1.32 mm Hg (CI, 0.12 to 2.52; *P *= 0.03) for systolic office blood pressure and 0.68 (CI, -0.13 to 1.49; *P *= 0.10) for diastolic office blood pressure. Furthermore, in the 199 patients with 24-h urinary sodium excretion below 200 mmol at baseline and at 5 and 10 weeks, the corresponding estimates were 0.88 mm Hg systolic (CI, -0.85 to 2.61; *P *= 0.18) and -0.39 mm Hg diastolic (CI, -1.53 to 0.75; *P *= 0.50). Finally, analyses of the office blood pressure (Table 8) and the 24-h blood pressure (Table 9) in previously untreated patients were also in agreement with the overall results of the trial, as shown in Tables 2 and 3, respectively.

**Table 8:** Office blood pressure on treatment with placebo or different daily doses of rostafuroxin in previously untreated patients

		Mean level ± SD at end of treatment period		Mean difference (95% CI)	*P*-values		
		
	N	Placebo	Rostafuroxin	Rostafuroxin minus placebo	Treatment effect	Carryover effect	Period effect
Systolic pressure, mm Hg							
0.05 mg	57	141.1 ± 11.8	145.8 ± 12.8	4.59 (1.43 to 7.74)	0.005	0.319	0.174
0.15 mg	41	140.3 ± 12.1	140.7 ± 13.8	0.26 (-4.10 to 4.61)	0.906	0.312	0.062
0.5 mg	38	142.1 ± 15.5	142.9 ± 13.4	0.72 (-4.01 to 5.45)	0.759	0.271	0.530
1.5 mg	41	140.9 ± 11.8	142.9 ± 12.6	2.02 (-1.46 to 5.51)	0.248	0.414	0.959
5.0 mg	48	139.1 ± 9.9	142.1 ± 12.9	2.89 (-0.54 to 6.31)	0.096	0.477	0.113
All doses	225	140.7 ± 12.1	143.0 ± 13.1	2.35 (0.71 to 4.00)	0.005	0.674	0.044

Diastolic pressure, mm Hg							
0.05 mg	57	87.4 ± 8.4	89.2 ± 8.5	1.78 (-0.45 to 4.01)	0.115	0.951	0.417
0.15 mg	41	88.0 ± 8.5	88.3 ± 9.9	0.29 (-2.30 to 2.88)	0.823	0.836	0.252
0.5 mg	38	87.8 ± 8.6	88.6 ± 8.2	0.80 (-2.61 to 4.21)	0.636	0.112	0.873
1.5 mg	41	88.0 ± 8.9	90.8 ± 6.4	2.82 (0.51 to 5.12)	0.018	0.344	0.191
5.0 mg	48	86.7 ± 8.0	88.4 ± 7.8	1.74 (-1.17 to 4.66)	0.235	0.510	0.173
All doses	225	87.5 ± 8.4	89.1 ± 8.2	1.53 (0.37 to 2.69)	0.010	0.714	0.033

Mean differences were estimated using analysis of variance with sequence, subjects nested within sequence, and period and treatment group entered as covariables. Abbreviations: N = number of patients; SD, standard deviation; CI, confidence interval.

**Table 9:** 24-h ambulatory blood pressure on treatment with placebo and different daily doses of rostafuroxin in previously untreated patients

		Mean level ± SD at end of treatment period		Mean difference (95% CI)		*P*-values	
		
	N	Placebo	Rostafuroxin	Rostafuroxin minus placebo	Treatment effect	Carryover effect	Period effect
Systolic pressure, mm Hg							
0.05 mg	55	131.9 ± 10.7	133.9 ± 11.5	2.02 (-0.68 to 4.73)	0.139	0.129	0.282
0.15 mg	39	130.9 ± 10.7	131.5 ± 11.8	0.51 (-2.59 to 3.60)	0.743	0.179	0.588
0.5 mg	36	135.2 ± 9.5	135.2 ± 9.4	0.04 (-3.38 to 3.46)	0.981	0.523	0.815
1.5 mg	40	135.3 ± 14.0	137.7 ± 11.7	2.39 (-1.20 to 5.99)	0.185	0.975	0.193
5.0 mg	44	133.7 ± 10.5	133.0 ± 12.2	-0.68 (-3.72 to 2.36)	0.654	0.831	0.749
All doses	214	133.3 ± 11.2	134.2 ± 11.5	0.91 (-0.45 to 2.28)	0.188	0.598	0.911

Diastolic pressure, mm Hg							
0.05 mg	55	80.4 ± 7.4	81.9 ± 7.8	1.53 (-0.22 to 3.28)	0.085	0.238	0.436
0.15 mg	39	81.9 ± 7.6	81.5 ± 8.1	-0.42(-2.80 to 1.97)	0.725	0.952	0.328
0.5 mg	36	85.6 ± 7.8	84.2 ± 9.1	-1.42(-3.77 to 0.94)	0.230	0.803	0.739
1.5 mg	40	82.5 ± 9.5	84.0 ± 7.7	1.52 (-0.42 to 3.47)	0.121	0.667	0.463
5.0 mg	44	82.4 ± 7.4	82.2 ± 6.9	-0.16 (-2.07 to 1.75)	0.867	0.719	0.927
All doses	214	82.4 ± 8.0	82.7 ± 7.9	0.32 (-0.57 to 1.22)	0.474	0.405	0.757

Mean differences were estimated using analysis of variance with sequence, subjects nested within sequence, and period and treatment group entered as covariables. Abbreviations: CI, confidence interval; N = number of patients; SD, standard deviation.

## Discussion

The primary objective of the double-blind OASIS HT trial was to identify the minimal daily dose at which rostafuroxin in patients with uncomplicated hypertension would reduce blood pressure significantly more than placebo. The primary endpoint was the reduction in systolic blood pressure defined as the average of 3 office readings with the patient in the sitting position. The key finding was that rostafuroxin compared to placebo did not reduce blood pressure at any dose or at all doses combined. Side-effects were rare and occurred with similar frequencies on placebo and rostafuroxin.

### Design of OASIS-HT

Phase 2 dose-finding trials commonly implement a parallel-group design, in which patients are randomized to incremental doses of the investigational drug and an untreated control group. The European Project on Genes in Hypertension [12-15] and several other studies [16,17] demonstrated that phenotype-genotype relations depend on host factors, such as sex and age, as well as on lifestyle, in particular salt intake as reflected by the 24-h urinary excretion of sodium. These reports highlighted the concept that phenotype-genotype associations can only be studied within a defined ecogenetic context. Furthermore, previous population studies demonstrated interaction between the plasma concentration of ouabain and the 24-h urinary sodium excretion in relation to systolic and diastolic blood pressures [18]. Cross-over studies compared to parallel-group designs, and ambulatory monitoring compared to the office measurement of blood pressure, reduce the variability in the estimated effects of any antihypertensive intervention and therefore allow economizing on sample size. These considerations explain the design of the OASIS HT trial, which is a combination of 5 concurrent cross-over studies, one for each dose of rostafuroxin to be studied, and the use of ambulatory monitoring to measure the secondary blood pressure endpoints.

### Clinical development of rostafuroxin as antihypertensive agent

In placebo-controlled phase-1 studies in healthy male volunteers (Sigma Tau, data on file), who received rostafuroxin in a daily dose ranging from 1 to 10 mg for up to 7 days, no clinically significant adverse effects occurred [4,6,19]. In particular, as in the present study, the compound had no influence on electrocardiographic measurements, including the RR, PR, QRS, QT and QT_c _intervals. After the initial dose, a few volunteers in the active-treatment and placebo groups had mild complaints, mainly headache, which all subsided uneventfully. Because of glucuronide hydrolysis, the parent compound was undetectable in urine, but the excretion of the urinary metabolite (PST2490) linearly increased with the orally administered dose.

In an uncontrolled phase-2 pilot study with forced titration, never-treated hypertensive patients received rostafuroxin during 3 sequential periods of 1 month in daily doses of 0.1 mg, 1 mg, and 5 mg, respectively (Sigma Tau, data on file [4]). Compared to baseline, the decreases in mean arterial pressure averaged 3.0 mm Hg (*P *= 0.039), 5.0 mm Hg (*P *= 0.007), and 5.0 mm Hg (*P *= 0.014), respectively. In a second study with double-blind design (Sigma Tau, data on file [4]), 42 and 21 patients with uncomplicated hypertension were randomized to 12 weeks of treatment with either rostafuroxin 0.5 mg/d or losartan 50 mg/d. The intention-to-treat analysis showed that at the end of follow-up blood pressure was slightly higher on rostafuroxin than losartan, but that the proportion of patients whose diastolic blood pressure dropped to 90 mm Hg or less was 38.0% in the 2 groups. Of the 42 patients randomized to rostafuroxin, 38 showed a decrease in systolic blood pressure [4]. All of these 38 patients had a sodium excretion below 210 mmol per day [4]. Whether salt intake explains the contradiction between this previous and the current study cannot be established with certainty. In the previous study (Sigma Tau, data on file [4]), mild adverse events were equally frequent (19.0%) on rostafuroxin and losartan, and with the exception of one case of gastritis on rostafuroxin, they were all considered to be unrelated to treatment. In agreement with these previous observations, the present study did not reveal any serious drug-related adverse event.

### Interpretation of the current results

In the present dose-finding study, rostafuroxin at any dose or at all doses combined did not lower blood pressure. In fact, compared with placebo, the office systolic blood pressure increased by 1.30 mmHg (*P *= 0.034) in all patients on rostafuroxin and by 2.72 mm Hg (*P *= 0.044) in those randomized to 0.05 mg/d. Several mechanisms might explain these negative findings. First, the large drop in blood pressure between baseline and the end of the first double-blind period (Figure 4) and the slight but significant period effect in the office blood pressure might have concealed a real antihypertensive effect of rostafuroxin. We therefore did an alternative analysis based on the first randomization to parallel groups. The results of this parallel-group analysis, in which we compared the blood pressure changes on placebo and on active medication from baseline to week 5, confirmed the double-blind cross-over analysis.

Second, previous studies demonstrated that the blood pressure lowering action of 0.5 mg/d of rostafuroxin occurred only at a 24-h urinary sodium excretion below 210 mmol. Sodium excretion at baseline averaged 152.8 ± 55.7 mmol/24 h (5-95th percentile interval, 69.4 to 221.0) and subsequently increased to 170.2 ± 74.4 mmol/24 h (5-95th percentile interval, 71.6 to 314.0) at 5 weeks and to 174.8 ± 73.8 mmol/24 h (5-95th percentile interval, 71.2 to 295.6) at 10 weeks. A 24-h urinary sodium excretion below 200 mmol was a positive selection criterion at the end of the run-in period [3]. However, of 386 analyzable patients, only 199 (51.6%) maintained a 24-h urinary sodium excretion below this threshold at both 5 and 10 weeks. Sensitivity analyses, in which the office blood pressure was adjusted for the 24-h urinary sodium excretion, or which were limited to patients with a 24-h urinary sodium excretion below 200 mmol throughout the study, confirmed the main results given in Table 2.

Finally, of the 410 analyzed patents, 193 (47.1%) had been previously treated for hypertension. Antihypertensive treatment potentially conceals the effects of mutated adducin and ouabain on blood pressure. A 4-week period was probably insufficient to wash-out all effects of prior treatment. However, for reasons of recruitment, it proved impossible to limit the recruitment to never-treated patients, as we did before [20]. For ethical reasons, we could not extend the wash-out beyond 4 weeks. However, sensitivity analyses in never treated patients also confirmed the main results presented in Tables 2 and 3.

## Conclusion

Rostafuroxin did not reduce blood pressure at any dose. Important secondary objectives of the OASIS-HT trial were to investigate the dependence of the blood pressure lowering activity of rostafuroxin on genetic variation in the enzymes involved in the synthesis and breakdown of ouabain and on polymorphisms in the adducin cytoskeleton proteins. The results of these analyses are beyond the scope of this report and will be reported elsewhere. In short, the pharmacogenomic study revealed that carriers of a genetic profile stated *a priori *and representing 23% of the patients randomized in OASIS-HT showed a significant decrease in blood pressure. Based on these preliminary pharmacogenomic results, we are now starting a more focused phase-2 trial comparing the effects of rostafuroxin and losartan in hypertensive patients with a specific genetic profile (EudraCT Number 2010-022073-34).

## Conflict of interests statement

The Studies Coordinating Centre (Leuven, Belgium) received financial support from Sigma-Tau for the scientific coordination of the OASIS-HT trial within the legal framework of Leuven Research and Development (http://www.kuleuven.be/lrd/about/mission.html). Giuseppe Bianchi is an advisor to the Prassis Research Institute (Settimo Milanese, Milan, Italy), which is affiliated with Sigma-Tau. Antonella Bachieri and Giovanni Valentini are employees of Sigma-Tau and Patrizia Ferrari is an employee of the Prassis Research Institute.

## Authors' contributions

JAS had full access to all of the data and takes responsibility for the integrity of the data and the accuracy of the data analysis. PF, LC and GB generated the hypothesis based on cell and experimental data and studies in hypertensive patients. GB and JAS conceived and designed the trial. KSS, JB, EdE, PWdL, MD, NG, AJ, PM, VM, YN, MS, CL, MT, AT, and KKJ acquired the clinical data. LC and NG coordinated the management of samples for centralized biochemical analyses. JB, PWdL, YN, and KKJ were national coordinators. LT and AB contributed to database management and the statistical analysis. LT did the statistical analysis at the independent statistical analysis center in Leuven. GV represented the sponsor, supervised the trial from an organizational point of view and coordinated obtaining the approvals from national and European Regulatory Authorities. JAS, LT and GB drafted the manuscript. All authors revised the manuscript for important intellectual content and approved the final version. GB and GV secured financial support from Sigma Tau Industrie Farmaceutiche Riunite.

## Appendix

### Participating centers

*Belgium*―H Celis (Leuven); *The Czech Republic*―R Cifková (Prague), J Filipovský (Pilsen), J Peleška, J Widimský Jr (Prague), M Souček, J Vítovec (Brno), J Špinar (Brno), V Stanek J Widimský Sr (Prague); *France*―A Fournier (Amiens); *Germany*―E Brand, SM Brand-Herrmann (Münster); *Ireland*―J. Barton, C. Kelly (Galway); *Italy *―G Basso (Schio), E Casiglia, V Tikhonoff (Padua), G Colangeli (Castelfranco), E Degli Esposti (Ravenna), N Glorioso (Sassari), P Manunta (Milan), M Timio (Foligno); *The Netherlands*―PW de Leeuw (Maastricht), M Krekels (Sittard); *Poland*―M Dłuzniewski (Warsaw), T Grodzicki (Krakow), A Januszewicz (Warsaw), K Kawecka-Jaszcz, A Olszanecka, K Stolarz-Skrzypek, M Klocek (Krakow), K Narkiewicz, B Wyrzykowski (Gdansk), W Piwowarska (Krakow), A Tykarski (Poznan); *The Russian Federation*―Y Nikitin (Novosibirsk), V. Milyagin (Smolensk), S. Nedogoda (Volgograd); *Slovenia*―J Brugljan, R Acceto (Ljubljana); *Spain*―P Armario (Barcelona), C Calvo-Gomez (Santiago de Compostela), JA Division-Garrote (Salud), A Coca-Payeras (Barcelona), F Hernández-Menárgez (Murcia), M Luque-Otero (Madrid), M Pascual-Izuel (Sagunto), A Pose-Reino (Santiago de Compostela), J Redón (Valencia); *Switzerland*―M Burnier.

### Coordination

*Scientific Coordinators*―J A Staessen, G Bianchi; *National Coordinators*―R Acceto (Slovenia), G Bianchi (Italy), E Brand (Germany), M Burnier (Switzerland), H Celis (Belgium); J Filipovský (Czech Republic), A Fournier (France), K Kawecka-Jaszcz (Poland), G McInnes (United Kingdom), Y Nikitin (Russian Federation), ET O'Brien (Ireland), J Redón (Spain); *Studies Coordinating Office*―T Kuznetsova, Y Jin, T Richart, JA Staessen, L Thijs (Leuven, Belgium); *Logistic Coordination*―A Bacchieri, G Valentini (Pomezia, Italy).
